# The Risk of Fatal Arrhythmias in Post-Myocardial Infarction Depression in Association With Venlafaxine

**DOI:** 10.7759/cureus.29107

**Published:** 2022-09-13

**Authors:** Sai Dheeraj Gutlapalli, Vamsi Krishna Lavu, Rana Abdelwahab Mohamed, Ruimin Huang, Shanthi Potla, Sushen Bhalla, Yousif Al Qabandi, Savitri Aninditha Nandula, Chinmayi Sree Boddepalli, Pousette Hamid

**Affiliations:** 1 Internal Medicine, California Institute of Behavioral Neurosciences & Psychology, Fairfield, USA; 2 Dermatology, California Institute of Behavioral Neurosciences & Psychology, Fairfield, USA; 3 Psychiatry and Behavioral Sciences, California Institute of Behavioral Neurosciences & Psychology, Fairfield, USA; 4 Internal medicine, California Institute of Behavioral Neurosciences & Psychology, Fairfield, USA; 5 Neurology, California Institute of Behavioral Neurosciences & Psychology, California, USA

**Keywords:** snri, venlafaxine, depression, post-myocardial infarction, arrhythmia

## Abstract

Venlafaxine is a second line anti-depressant and the most commonly used in the treatment of selective serotonin reuptake inhibitor nonresponders in major depression; due to its effects on the noradrenergic and serotonergic systems as a serotonin and norepinephrine reuptake inhibitor, there has been considerable apprehension regarding its use in patients with cardiovascular diseases, particularly post-myocardial infarction depression, some of the feared adverse effects include QT prolongation, arrhythmias including torsades de pointes and sudden cardiac death. We tried to resolve the facts regarding the risks associated with venlafaxine use in cardiac patients. We have reviewed all the relevant information up to May 2022 regarding the risks of venlafaxine use in cardiovascular disease, particularly with a focus on post-myocardial infarction depression, and gathered around 350 articles in our research and narrowed it down to 49 articles. The database used was PubMed and the keywords used were venlafaxine, arrhythmia, major depression, post-myocardial infarction, and ventricular tachycardia. We carefully screened all relevant articles and found articles supporting and refuting the effects of venlafaxine in increasing cardiovascular morbidity and mortality. We have concluded that there is a significant variability due to confounding factors affecting individual cases. Overall there is no increased arrhythmia risk in comparison with other anti-depressants except in high-risk cases such as with pre-existing cardiovascular disease, certain genotypes, and other co-morbidities. Any patient with a high risk of arrhythmias due to any etiology should receive a screening electrocardiogram before venlafaxine prescription for baseline QT interval and periodically while on therapy to check for changes. We encourage further research, including randomized clinical trials and post-marketing surveillance regarding the use of venlafaxine in high-risk cases such as patients with multiple co-morbidities, elderly patients, or patients with certain genotypes.

## Introduction and background

Major depression is a serious co-morbid condition in patients with cardiovascular disease (CVD), it can be triggered due to myocardial infarction (MI) and pre-existing major depression can be worsened by MI [1]. The association between causation of CVD including coronary artery disease (CAD) and incidence of mental disorders like major depressive disorder (MDD) and anxiety is bi-directional; there is a high frequency of anxiety, major depression, and panic attacks in CVD patients [2-4]. Factors like anxiety, depression, and low socioeconomic status are also directly associated with worse prognoses in patients with pre-existing CAD [5]. More than half the patients with CAD suffer from depressive symptoms and around twenty percent have major depression, Major depression is an independently associated risk factor for increased cardiovascular morbidity and mortality [6,7].

Selective serotonin reuptake inhibitors (SSRIs) and serotonin-norepinephrine reuptake inhibitors (SNRIs) are the preferred pharmacological treatments in such patients [2]. Electrocardiogram (ECG) monitoring is always recommended to check for corrected QT interval (QTc) prolongation in high-risk patients such as elderly patients or patients with multiple co-morbidities [2]. SSRIs and SNRIs do not have higher efficacy than older anti-depressants like tricyclic anti-depressants (TCAs) but have a favorable side effect profile [8]. Venlafaxine, an SNRI, is known to increase heart rate (HR), blood pressure (BP), and QTc prolongation at higher doses [2].

We know that depression and anxiety disorders are associated with lower adherence to treatment, frequent hospitalizations, poor overall functional status, and increased mortality in patients with heart failure (HF) [9]. Studies showed an increase in long-term survival in CAD patients who were treated for depression; nearly half of the patients had remission in two months on monotherapy with an SSRI and the non-responders to SSRIs treated with venlafaxine had a remission rate of 25% [4]. It is known that depression is a predictor of new and recurrent atrial fibrillation, stress and anxiety have also been shown to trigger arrhythmias [5]. While prescribing anti-depressants, we must always consider drug-drug interactions and QTc prolongation in patients with any form of CVD, particularly HF, MI, and arrhythmias [5]. We know that risk of arrhythmia and sudden cardiac death (SCD) is higher in depressed patients even without any history of cardiovascular issues [6]. The cardiovascular side effects of anti-depressants range from tachycardia, bradycardia, hypotension, hypertension (HTN), ECG changes, electrolyte imbalances, decreased cardiac output, arrhythmias, and SCD [6]. We know that SNRIs including venlafaxine are similar in terms of mechanism, efficacy, and side effects profile to SSRIs [6]. Rarely, Venlafaxine has been shown to cause tachycardia, angina, extrasystoles, and congestive HF (CHF) in some patients [10]. Overall the evidence shows that pharmacotherapy and cognitive behavioral therapy (CBT) are both effective in the treatment of depression in patients with CVD [10].

The focus of our research is on the cardiovascular adverse effects of venlafaxine in post-MI depression. In light of the global burden of major depression in patients with CVD, we know that the majority of SSRI non-responders are offered SNRIs like venlafaxine, which may include a patient population of more than 300,000. We performed a risk and benefit analysis of venlafaxine treatment by focusing on cardiovascular morbidity and mortality in this study. The relevant data for our literature review was gathered from the PubMed database, five keywords were used "arrhythmia," "major depression," "post-myocardial infarction," "venlafaxine", and "ventricular tachycardia". The search was performed using the MeSH strategy. We manually screened and included all the relevant articles we could find from inception till May 20th, 2022. All data were sourced from PubMed.

## Review

Cardiovascular disease and major depression 

By 2030, depression will become one of the biggest causes of disease burden worldwide [7]. World Health Organization (WHO) reports that ischemic heart disease is the world’s leading cause of death, while depression and CAD are the two major causes of years lost due to disability by 2020 [4,6]. The risk of mental health disorders is higher in patients with pre-existing CVD, and there is a high frequency of psychiatric illnesses like depression, anxiety, and panic attacks increasing the risk of new-onset CVD in healthy patients and worsening morbidity in patients with pre-existing CVD [1-3,5,6].

MDD may be triggered by MI or may precipitate it and up to half of CVD patients suffer from depressive symptoms and at least 20% have MDD [1-3,5,6]. The association between CVDs like CAD and mental health disorders like MDD is multi-directional [2,4]. According to a study, as high as 65% of patients with MI have a psychiatric disorder, most commonly depression and/or anxiety [4]. Studies by Khawaja et al. have shown up to 40% of patients with CAD have a diagnosis of major depression in their lifetime [4]. In another study, it was shown that nearly 25% of patients post-coronary artery bypass graft surgery (CABG) had depression [4]. A history of MI is an independent risk factor for depressive symptoms in hospitals and patients with a history of depression were four times more likely to have an acute MI at follow-up compared to controls [4]. Patients with depression during hospital stay were more likely to have depressive symptoms after discharge [4]. Depression is independently associated with an 18% increased risk of developing HF in the next seven years and exacerbation of pre-existing HF symptoms [9].

The focus on depression in pharmacotherapy started in the 1950s [8]. There are almost 20 different anti-depressants available worldwide [8]. Depression and anxiety disorders are associated with three times lower adherence to treatment, double the rate of diabetes, unhealthy lifestyle, hypercholesterolemia, malnutrition, smoking, obesity, drinking, sleep disorders, physical inactivity, substance abuse, frequent hospitalizations, poor overall function, and increased mortality in patients with HF when compared to patients without HF [4,5,9,10]. 

Studies showed that MI is often preceded by a prodrome of minor depression with a follow-up time of around 4.5 years [4]. Studies showed higher rates of arrhythmias, angina, recurrent infarction, ischemia, and CHF during the first hospital stay and high rates of readmissions from these issues in depressed patients compared to non-depressed patients, depression in CVD doubles the morbidity and mortality; it is also a predictor of post-MI cardiac mortality and hospitalization for CHF which is 2.8 times higher than in non-depressed patients a year after MI [4,7]. 

Individuals with type-D personalities have been found to have an increased incidence of depression and anxiety [5]. In addition, the risk of arrhythmia and SCD is higher in depressed patients even without any history of CVD [6]. Roughly eight million people in America are suffering from HF as of the year 2020, with mortality reaching 50% within five years of being diagnosed, up to half of these patients have co-morbid depression, and treating MDD is essential to decrease hospitalizations [5,6,9].

Mechanisms linking heart disease and depression 

According to the American Heart Association, acute coronary syndrome (ACS) increases the risk of MDD by two to three times compared to the general population and vice versa; prognosis is worsened by dysregulation of serotonin, norepinephrine, dopamine systems, sympathetic overactivity, and parasympathetic under-activity with ACS and co-morbid MDD increasing CVD morbidity and mortality [6,7].

Furthermore, depression leads to higher levels of inflammation, which plays a key role in connecting the outcomes of HF to psychiatric symptoms by affecting ventricular remodeling and promoting fibrosis, causing cardiac dysfunction [9]. Autonomic dysregulation associated with depression leads to arrhythmias and adverse cardiac remodeling, and heart rate variability (HRV) is reduced in MDD, increasing the risk for autonomic dysregulation [9]. In CAD, depression is associated with endothelial dysfunction, elevated C-reactive protein, platelet dysfunction, and impaired flow-mediated dilation leading to accelerated atherosclerosis and plaque formation [4,9].

HRV and baroreflex sensitivity (BRS) are useful tools to understand these phenomena, and along with psychopharmacological treatments, a non-invasive breathing technique known as HRV biofeedback (HRVB) can increase the parasympathetic tone [7]. HRV is a reliable predictor of arrhythmias along with ECG markers; some patients may develop arrhythmias even with normal ECG; hence HRV abnormality is more predictive of arrhythmias in certain patients [6]. A high HRV is been shown to be protective against HF, MI, and fatal arrhythmias [6]. HRV is mainly controlled by cardiovagal activity, and up to 70% of cardiovagal tone is dictated by the arterial baroreflex mechanism [7]. Reduced HRV and BRS are associated with acute stress, MI, ventricular fibrillation after MI, chronic CHF, dilated cardiomyopathy, hypertension, and increased risk for SCD [7]. A decrease in HRV and BRS is seen with depression, and HRVB may be a useful new add-on to current psychopharmacological treatments for depression in CVD patients [7].

**Figure 1:**
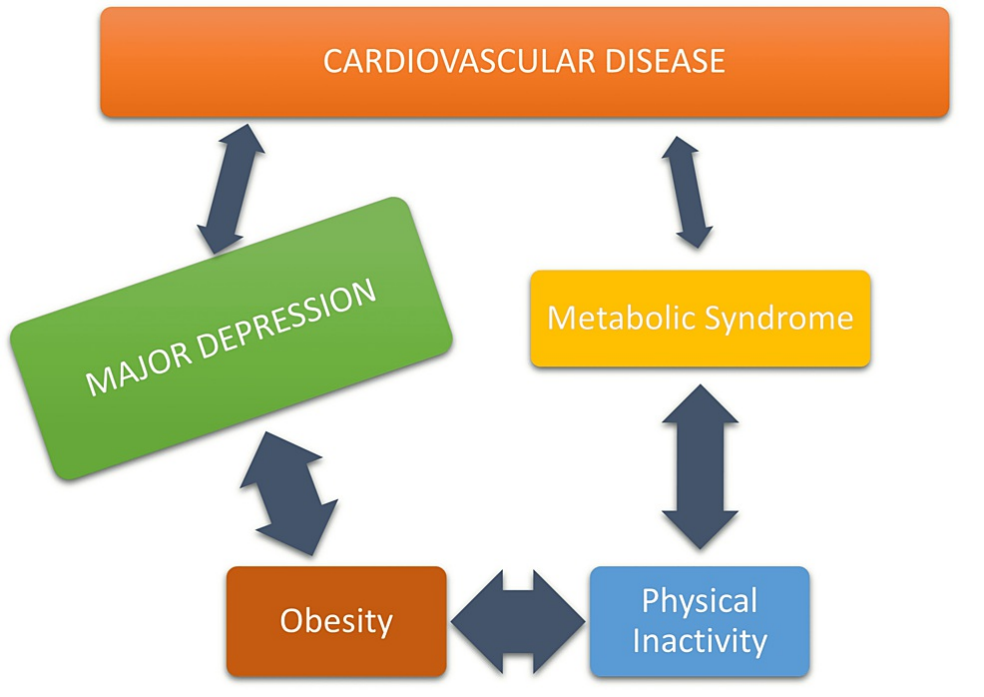
The multi-directional web of causation.

The prevalence of CHF is among more than 6.6 million Americans and fifteen million patients in the EU region, totaling around 25 million patients in the US, UK, and EU combined [10]. Depression increases the risk of CHF by three times that compared to the general population [10]. Studies have shown that 20% of patients who have a MI or undergo CABG have major depression [10]. Almost 15% of the elderly in America are now taking an anti-depressant [11]. The lifetime occurrence of depression is almost 20% worldwide [12]. A million Americans suffer from ACS every year and a half have prior depression [13]. ACS is an umbrella term for both MI and unstable angina (UA) which are important life-threatening events in CVD [13]. Studies have shown that depression can be stable for years post-MI in untreated individuals [13]. In addition, patients with ACS in-hospital with major depression had been depressed for over a month in almost 95% of the cases, and around 60% have been depressed for at least six months [13]. The chain of association linking CVD and MDD is shown in Figure 1.

Psychological stress and other mental health disorders associated with CVD

Anxiety is an independent risk factor for CVD [13]. Almost a quarter of the patients with CVD have GAD and almost 50% of patients post-MI have acute anxiety; persistent anxiety up to two years post-MI is common [13]. Patients with CAD have also been noted to have up to 40% higher rates of anxiety compared to the general population [13]. Anxiety has been shown to decrease anti-depressant efficacy in patients by its effects on the adrenergic system [9].

Childhood and adolescent stress levels are associated with the development of CVD in later decades [5]. Chronic stress in association with the endocrine, inflammatory and autonomic nervous systems has been shown to increase the risk of cardiac events in patients with CVD [5]. Cognitive deficits are observed in almost half of the patients with cardiac disease, especially patients with severe cardiovascular insufficiency or a history of open-heart surgery, and the risk of dementia is doubled in patients with HF [5]. Studies have shown some patients who developed post-traumatic stress disorder (PTSD) post-MI and depressed CVD patients should also be screened for co-morbid dementia [5,12]. Moreover, functional heart complaints like chest pain or palpitations sometimes don’t have an organic cause confounding clinicians, and these complaints are psychogenic in almost 15% of cases, and more than 50% of functional heart complaints are seen in patients with psychiatric disorders [5].

Depressed patients have higher levels of corticotropin-releasing factor in their cerebrospinal fluid, thus increasing corticosteroids and accelerating atherosclerosis, hypertriglyceridemia, hypertension, and hypercholesterolemia [4]. Depression has been shown to decrease HRV and increase QT variability leading to arrhythmias, and lower HRV has been shown to increase mortality after MI [4,6]. Social support is found to be protective both in the prevention and progress of CVD [5]. Palliative care can be considered in some patients with highly progressive heart disease [5].

Acute psychological stress has been shown to trigger ACS, ventricular tachycardia, and stress-induced cardiomyopathy [5]. Supraventricular tachycardia causes significant impairment in quality of life and sometimes can be confused with panic attacks in patients with anxiety [5]. Depression is a predictor of new and recurrent atrial fibrillation, stress and anxiety also trigger arrhythmias [5].

Factors affecting cardiac conduction 

**Figure 2:**
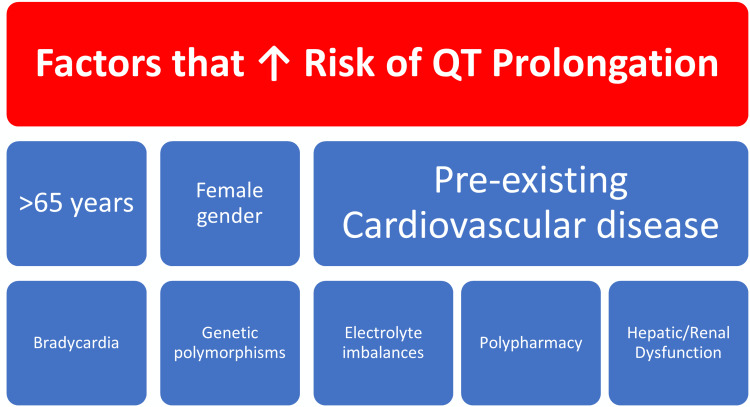
List of the factors increasing the risk for QT prolongation.

The QT interval in ECG is predictive of the development of arrhythmias, the normal corrected QT interval (QTc) length is approximately 400 milliseconds (ms) with slight variation between men and women, and QTc of more than 500 ms may lead to torsades de pointes (TdP), which is a dangerous and potentially fatal polymorphic ventricular tachyarrhythmia [6]. Important factors risk factors for QTc prolongation are age ≥ 65 years, bradycardia, myocardial hypertrophy, female gender, hypomagnesemia, hypokalaemia, pre-existing heart disease, high plasma drug concentrations, reduced drug clearance due to renal/hepatic failure, genetic ion channel polymorphisms, variations in cytochrome enzymes leading to slower drug metabolism and congenital long QT syndromes [6]. The factors increasing arrhythmogenic risk are shown in Figure 2.

Global financial burden

In America, roughly $50 billion a year are spent on the medical expenses associated with CHF alone and the cost of care for patients with co-morbid depression in CHF is 30% higher, and treatment of depression also consumes 1% of the overall gross domestic product of Europe [7,10].

Exercise in CVD and MDD

Regular exercise of 30 mins a day is equally effective as sertraline in some studies as an anti-depressant therapy and is shown to decrease all-cause mortality by 25% in CVD, post-MI depression during cardiac rehabilitation can be treated effectively using a combination of psychopharmacology and exercise [4,5,12,13]. Exercise training in cardiac rehabilitation in depressed patients is associated with an almost 75% reduction in mortality when compared to patients who did not exercise and patients who exercised regularly had a 50% reduction in adverse events, non-fatal reinfarction, and reduced mortality two years after MI [4,5,12,13].

Additional important points to consider in CVD and MDD 

The Johns Hopkins precursor study reported that depression is an independent risk factor for subsequent development of CVD and MI in the long term; patients with depression were associated with as much as 60% higher risk of CVD [13].

Pre-existing and post-MI depression significantly impaired medical outcomes in comparison to non-depressed patients with a higher incidence of recurrent cardiac events in-hospital and greater mortality [13]. In a meta-analysis of 6000 patients, it was shown that post-MI depression was associated with a 2.6 times higher risk of mortality in the following year and almost doubled the rate of recurrent MI compared to non-depressed patients [13]. Patients with treatment-resistant post-MI depression have the highest risk of adverse outcomes [13].

Severe anxiety is a stronger risk factor for cardiac disease than MDD; during acute CVD events and post-MI, anxiety was associated with significantly higher short- and long-term morbidity and mortality [13]. Patients with depression post-MI and HF have a lower motivation to follow a healthy lifestyle and are less likely to complete cardiac rehabilitation [9,13].

The prevalence and severity of depression post-MI correlated with the level of cardiac dysfunction and the likelihood of CHF; higher rates of depression were associated with lower left ventricular ejection fraction (LVEF) 3-12 months post-MI [10]. It has also been observed that patients with LVEF < 30% post-MI had a 4.46 odds ratio of being depressed compared to the patients who had LVEF > 60% (preserved LVEF), depression was associated with two-fold high mortality in CHF [10].

Electroconvulsive therapy (ECT) is the most effective treatment for depression in up to 80% of patients; the response rate is four times higher than pharmacotherapy, “cardiac-modified ECT protocol” can be used in patients with cardiac disease [10].

Cognitive behavioral therapy (CBT) is a very effective treatment for depression and anxiety with HF and improves the quality of life in patients post-MI, pharmacotherapy is more effective in CAD and other CVDs; almost 30% of the deaths each year happen due to heart disease worldwide, and 30% of patients with CAD or HF have symptoms of depression/anxiety, while 20% have major depression [4,9,13,14].

Anxiety and depression in ACS increase the risk of adverse events in-hospital and long-term cardiac morbidity and mortality post-ACS, noradrenergic hyperactivity and serotonergic dysfunction lead to poor outcomes due to negative stress caused by catecholamines on blood vessels, heart, platelet dysfunction, inflammation, decreased HRV and endothelial dysfunction all of which are important mechanisms linking ACS, CAD, and MI with depression/anxiety [4,9,13,14].

Venlafaxine and CVD 

Venlafaxine is an SNRI and has dual serotonergic and noradrenergic activity, it is one of the most frequently prescribed anti-depressants in SSRI non-responders, many of these patients have co-morbid cardiovascular disease [15]. Noradrenergic reuptake inhibition is attributed to its effectiveness at doses higher than 150 mg/day [15].

Venlafaxine treatment and adverse effects

The guidelines in Canada recommend the use of venlafaxine, bupropion, and mirtazapine as first-line drugs along with SSRIs instead of the second line, and American guidelines advise venlafaxine as a second-line drug in SSRI non-responders [11]. SSRIs are first-line drugs for depression post-MI and chronic CVDs, venlafaxine is the second line due to its effects on blood pressure; it is well tolerated with few anticholinergic side effects, no effects on cardiac conduction, minimal interaction with cytochrome CYP450 System in usual doses and can be safely given in hepatic dysfunction [4,5,12]. Evidence shows that pharmacotherapy, ECT, and CBT are all effective in the treatment of depression in patients with CVD particularly CHF [10]. Delay in effect is a major issue with anti-depressant therapy; remissions in mins, hours, or days are preferable to weeks [8]. Normal variations of moods are sometimes instantaneous compared to therapy, even ECT and sleep deprivation work faster than anti-depressant therapy [8].

**Figure 3:**
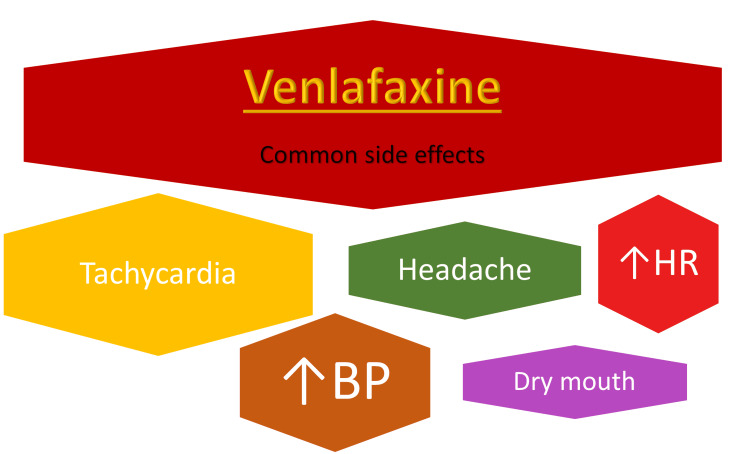
The most common cardiovascular side effects of venlafaxine. Heart Rate (HR); Blood Pressure (BP)

Around 40% reduction in re-infarction and cardiac mortality was shown in post-MI depression with CBT and pharmacotherapy over a follow-up period of 29 months compared to CBT alone [13]. SSRIs and SNRIs have a significantly lower cardiovascular and sexual adverse effect profile than older anti-depressants and do not prolong QTc at therapeutic doses, ECG monitoring is always recommended in patients at risk for QTc Prolongation [2,6,8,12-14]. A loss of bone mineral density (BMD) of up to 6% is seen with SSRIs, loss of BMD is not evident with venlafaxine [12]. Venlafaxine use has progressively increased in SSRI non-responders since it was first introduced in 1994 and venlafaxine-related mortality per million prescriptions has been consistently higher than SSRIs [16]. In a study, the median ingested dose of venlafaxine of 1500 mg resulted in tachycardia in 54% of patients and mild HTN in 40%, no arrhythmias were observed [17]. The dose-dependent increase in HR observed with venlafaxine may lead to an over-correction of the QT-interval using Bazett’s formula, and Bazett’s formula is known to be inaccurate outside of the HR range of 50-70 bpm [17]. Extremely rare cases of drug-induced interstitial pneumonitis and acute cardiomyopathy have been observed with venlafaxine toxicity due to genetic variations in drug metabolism and drug-drug, drug-herb interactions [18]. The common adverse effects are listed in Figure 3.

Venlafaxine contraindications and regulation

In a study of more than 200,000 patients with depression and/or anxiety over an average follow-up of 3.3 years, the use of venlafaxine was not associated with an increase in the risk of SCD compared to SSRIs [19]. In the UK multiple studies reported that venlafaxine was associated with higher mortality in overdose compared to SSRIs due to fatal arrhythmias by precipitating cardiac ischemia through a rise in BP and HR [19]. Between 2004 and 2006, venlafaxine prescriptions were restricted to specialists in the UK by regulators and contraindicated in patients with heart disease, HTN, and electrolyte imbalances due to the risk of fatal arrhythmias [16,19].

Venlafaxine and metabolic syndrome

Metabolic syndrome (MetS) affects a quarter of the world population, and 60% of patients with psychiatric disorders die from causes like CVD which are co-morbid with MetS, depression has a bidirectional relationship with MetS and obesity [20]. MetS increases the risk of QTc prolongation [20]. In animal studies venlafaxine increased the risk for central obesity, MetS, lowered ventricular fibrillation (VF) threshold, and shortened restoration time of sinus rhythm in combination with a high-fat-fructose diet; it also blocked fast inward sodium currents in ventricular myocytes leading to QRS prolongation [20].

Venlafaxine mechanism of action regarding toxicity, genetics, and effects on cardiac conduction

Behlke et al. reported that venlafaxine did not prolong QTc [15]. QTc is a proxy marker for drug-induced SCD risk [15]. Preclinical studies of venlafaxine showed no significant QTc prolongation [15]. Clinical studies haven’t been as conclusive for venlafaxine and suggest QTc prolongation may occur at higher rates than previously expected due to cytochrome enzyme CYPD26 variations and drug-drug interactions [15]. Drug-induced QTc variations are significantly influenced by the choice of the formula used to calculate QTc [15]. 

At therapeutic levels, venlafaxine has a thirty times higher affinity for serotonin receptors than noradrenaline receptors [20]. Venlafaxine may cause mild tachycardia, headache, dry mouth, increased HR and BP, decreased HRV, QTc prolongation, and TdP at higher doses by blockage of cardiac sodium channels, it acts like an SSRI at lower doses but is more efficacious at higher doses, so when it is prescribed in treatment-resistant depression, PTSD, generalized anxiety disorders (GAD), panic disorders close monitoring is advised to prevent adverse cardiac events like tachyarrhythmias, hypertensive crisis, angina, extra-systoles, CHF and SCD [2,6,8-10,15,16,20-22]. Inhibition of cardiac and vascular Ca2+, K+, and Na+ channels by SSRIs and SNRIs may be associated with cardiac adverse events in susceptible patients [23]. Nearly 75% of drug-induced QT prolongation events were caused by either antibiotics or anti-depressants in clinical practice, and 40% of patients with SCD have underlying ion channelopathies increasing the arrhythmogenic risk [24]. In patients taking anti-depressants factors other than QTc prolongation may be involved with arrhythmias including pre-existing heart disease, damaged myocardium, ventricular ectopic beats, and predisposition to the “R on T” phenomenon [25].

In cases of anti-depressant-induced arrhythmias, ECG markers like T wave alternans, QT dispersion, and the emergence of the Brugada pattern are important in differentiating causation [25]. Due to the risks of tachycardia and a rise in BP venlafaxine should be avoided in patients with HTN [25]. A study that determined the hazard ratios for TCAs, SSRIs, and SNRIs in association with emergency department and inpatient admissions for ventricular arrhythmias and SCD observed no increased risk for venlafaxine compared to other anti-depressants [26].

In a study regarding drug-induced long QT syndrome (LQTS), drug-induced Brugada syndrome, and drug-induced short QT syndrome, SCD still accounted for approximately half of all deaths from CVD, and ventricular fibrillation or tachycardia accounted for 75% of all the cases of SCD outside hospitals, this number has come down to 30-40 % in recent years due to decrease in CAD mortality [27]. An important predictor of SCD is drug-induced pro-arrhythmia [27]. Amplification of the spatial dispersion in repolarization of the ventricular myocardium is an important arrhythmogenic etiology in both acquired and congenital LQTS [28]. LQTS are subdivided into 10 genotypes with seventeen different genes coding for cardiac ion channels [28]. Acquired LQTS is due to drugs, cardiomyopathies, and other cardiac diseases [28]. In a study, a single polymorphism associated with predispositions to arrhythmias was present in more than 1% of the total population, many different types of such variations can exist and affect a greater percentage of the population than previously anticipated [28]. A change of medication is always advised in patients where QTc > 500ms [28].

The cardiac arrhythmia suppression trial was the first to suggest a link between anti-depressants and arrhythmias based on sodium channel blocking properties [29]. Evidence suggests that up to 15% of individuals who develop TdP after being exposed to drugs that prolong QTc have been associated with congenital LQTS [29].

Atrial fibrillation is so common that it is very difficult to determine if it is incidental or drug-induced and drugs are the most common cause of acquired LQTS [30]. In a study, 12.5% of patients with major depression treated with venlafaxine had new onset hypertension attributed to the drug [30]. Venlafaxine and TCAs both block the noradrenaline reuptake, which may be a contributing factor to the higher incidence of arrhythmogenicity and cardiotoxicity of venlafaxine compared to SSRIs [25,31,32].

In a meta-analysis by Cao et al., more than 2.6 million participants in around 3400 studies were analyzed, and it was concluded that anti-depressant did not increase the risk of ventricular tachyarrhythmias and SCD, but there was a significant association between anti-depressants and atrial fibrillation [33].

In a study by Nezafati et al., anti-depressants were divided into three groups based on their cardiovascular effects profile, group one - safe, group two - neutral including SNRIs, and group three - harmful [34]. In a study by Wiśniowska et al., they concluded that drug-drug interactions exhibit a high degree of variability in the general population making it essential for ECG monitoring in all high-risk patients taking drugs with possible QTc prolongation [35].

Genotyping patients for high-risk alleles is a useful strategy in clinical practice before prescribing anti-depressants known to prolong QTc; the decreasing costs of genotyping in the future is also a key point in this regard its implementation in clinical practice; it is also essential to decide If benefits outweigh the risks [36,37]. There was no difference between the incidence of MI, atrial fibrillation, and HF across the different types of anti-depressant classes [38]. Studies have shown that a serotonin transporter polymorphism was associated with an increased risk of premature MI inpatient, anti-depressants with a high affinity for serotonin transporter decreased the risk for MI [38].

Venlafaxine in elderly

In a large nationwide study in America, it was observed that around 540,000 elderly patients had been prescribed at least one anti-depressant, and a total of 17 million anti-depressant prescriptions were issued from 2008 to 2013, the majority of these prescriptions were SSRIs and SNRIs, and 48% of the prescribed anti-depressants have a known risk of TdP, 26% had a possible risk of TdP, 21% had a conditional risk of TdP [39].

In the elderly, there are concerns it may cause QTc prolongation, especially at high doses [15]. Venlafaxine has a moderate risk of QTc prolongation and TdP usually in the presence of multiple risk factors like CVD, bradycardia, and polypharmacy [40,41]. There have been cases of worsening HF after venlafaxine therapy in previously stable patients due to an increase in norepinephrine levels [42]. The advantages of venlafaxine for treatment in the elderly are low-cost, effectiveness, once-daily dosing by extended-release formula (XR), and very few drug-drug interactions [15]. Studies show that venlafaxine extended-release (XR) at 12-14 weeks shortened the QT interval in the elderly population and lowered the risk of HF in comparison to sertraline, serum drug concentrations did not correlate with QTc changes, and there was no effect on cardiac disease, depression or gender on these finding [9,15]. Venlafaxine can be used in the elderly without regular ECG monitoring at therapeutic doses, and it can be remotely initiated across telehealth platforms without the need for a baseline in-person ECG [15]. 

Venlafaxine metabolism and risk of overdose

Even though venlafaxine has a benign cardiac adverse effect profile, the rare cardiotoxicity may be due to dose-dependent effects on fast conducting cells in the ventricles by binding to the resting sodium channels, which is a different mechanism to TCAs [25,43,44]. Venlafaxine overdose in monotherapy is not likely to cause cardiac adverse events, QTc prolongation, or fatal arrhythmias except in cases of ingestions of >8 g or combined with TCAs [16,17,45]. Drug-induced LQTS due to venlafaxine has been observed in patients with hypomagnesemia and hypokalaemia [46]. Letsas et al. reported a case of a 60-year-old patient in-hospital with QTc prolongation normalizing after venlafaxine discontinuation [47].

**Figure 4:**
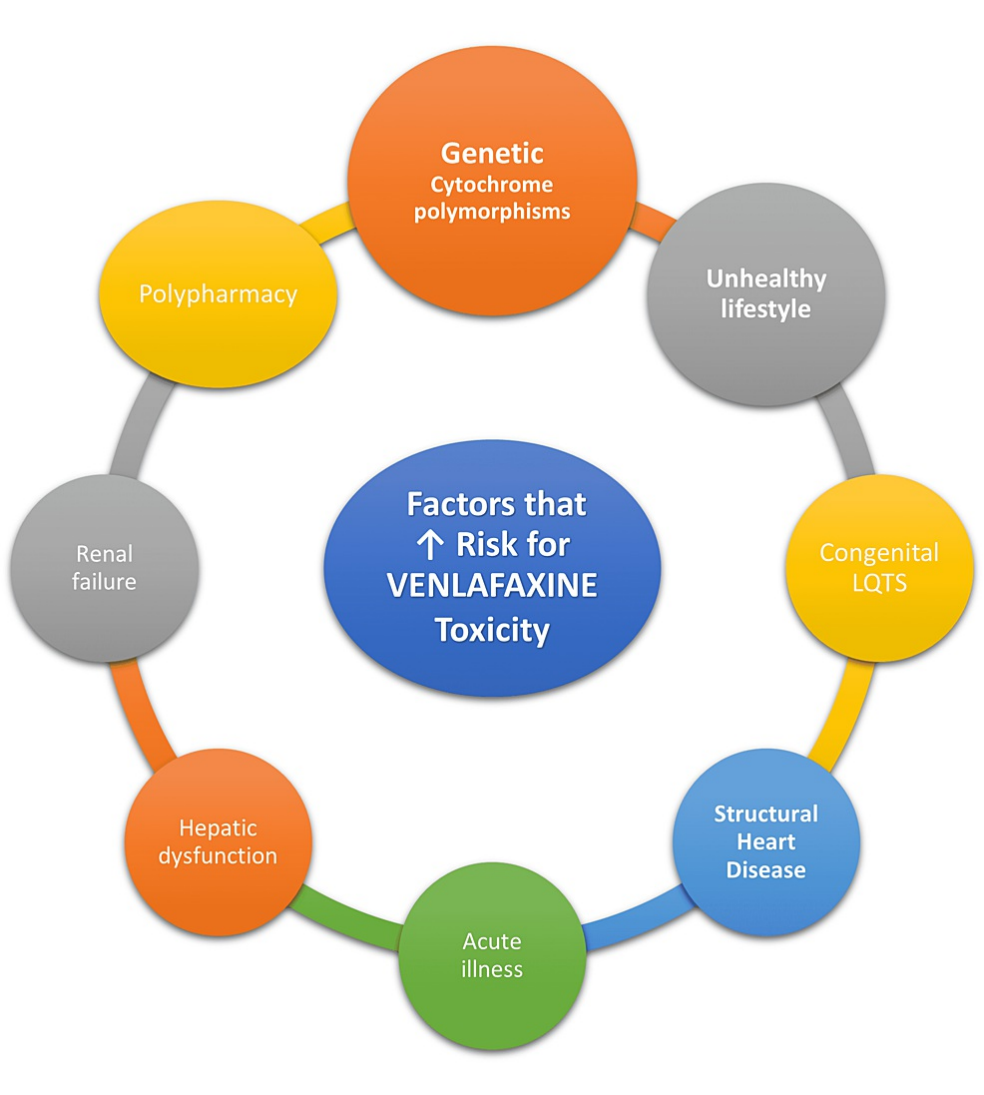
Factors associated with an increased risk of venlafaxine toxicity. Long QT Syndrome (LQTS)

According to the WHO, 72% of people with depression have at least one chronic physical condition; drug-drug interactions due to polypharmacy are a serious risk in these patients, these are divided into pharmacodynamic (PD) and pharmacokinetic (PK) interactions; in PD interactions one drug alters the function of another drug, in PK the metabolism, absorption, distribution, and elimination are affected by the other drug, the majority of these interactions are PK type and are frequently associated with genetic cytochrome polymorphism [48]. The hepatic cytochrome P450 system (CYP) is important for anti-depressant metabolism, there are more than fifty CYP isoenzymes, but only six play a major role in metabolizing anti-depressants, including CYP3A4 and CYP2D6; hence polymorphism in these enzymes or drugs inhibiting them can lead to poor metabolism and toxic levels of the drug in serum of certain patients [48]. The major active metabolite of venlafaxine is desvenlafaxine (O-desmethylvenlafaxine) after metabolism through CYP2D6, 45% of it is eliminated renally, Venlafaxine is metabolized to a minor extent by CYP3A4, but some studies have also shown that even a ten-fold rise in drug concentration did not correlate with QTc changes [15,48]. The simpler metabolism of venlafaxine compared to other anti-depressants makes it more favorable in the context of CYP-associated drug interactions [48]. Improvement in survival is seen in CVD patients with depression receiving pharmacotherapy, half the patients had remission in two months with SSRIs, and non-responders with severe illness treated with venlafaxine had a remission rate of 25% [4,14,19,49]. In a US study, it was observed that in 5510 overdoses due to venlafaxine alone only twelve were fatal, a cardiac disturbance was noted in less than 2% of the cases and fatality was 0.22% [49]. In a nationwide study from Denmark, it was observed that venlafaxine had the lowest odds of out-of-hospital cardiac arrest compared to other anti-depressants [49]. The factors known to be associated with an increased risk of venlafaxine toxicity are shown in Figure 4.

## Conclusions

After reviewing 49 studies specifically related to the context of arrhythmogenic risk of venlafaxine in patients post-myocardial infarction with major depression, we have concluded that venlafaxine does not pose an excess risk in comparison to other anti-depressants, but a careful history must be taken by the clinician regarding cardiovascular, thyroid, genetic, cerebrovascular, social including substance abuse, alcohol, smoking and any other factors which may potentially affect the metabolism of venlafaxine. Independent factors like metabolic syndrome, diabetes, and obesity also predispose to a higher risk of adverse effects. Genetic ion channelopathies and polypharmacy are also important deciding factors while considering the use of venlafaxine, CBT, and heart rate variability biofeedback may be tried before or in addition to prescribing pharmacotherapy in high-risk patients. Any patient with cardiovascular disease should have a screening ECG to assess baseline QT interval and periodic ECGs while on venlafaxine therapy as a safety check. It is essential to treat co-morbid psychiatric illness in patients with CVD to achieve positive outcomes, and venlafaxine is one of the key anti-depressants used in SSRI non-responders making it one of the most commonly prescribed drugs worldwide due to its noradrenergic effects may pose a risk to susceptible patients; therefore great care must be taken to prevent drug-drug interactions and screen for potential patient factors which can worsen prognosis in venlafaxine use. Overall it is a safe drug, with a mostly benign adverse effects profile, and can be used extensively in the general population. We suggest further research, including large-scale clinical trials, post-marketing surveillance, and pre-prescription genetic testing is encouraged regarding venlafaxine use in high-risk patients.
